# Retraction Note: breast cancer surgery in elderly patients: postoperative complications and survival

**DOI:** 10.1186/1471-2482-15-2

**Published:** 2015-01-14

**Authors:** Nicola Rocco, Corrado Rispoli, Giuseppe Rengo, Gennaro Pagano, Rita Compagna, Michele Danzi, Antonello Accurso, Bruno Amato

**Affiliations:** Department of Biomedical, Surgical and Dental Sciences, University of Milan, Milan, Italy; Department of General Surgery, Cardinale Ascalesi Hospital - ASL NA1, Naples, Italy; Department of Translational Medical Sciences, University “Federico II” of Naples, Naples, Italy; Department of General, Geriatric, Oncologic Surgery and Advanced Technologies, University “Federico II” of Naples, Naples, Italy

This article [1] has been retracted by the authors due to significant overlap with a previous publication [2]. Giuseppe Rengo and Gennaro Pagano were not involved in the study and were introduced into the author list by the corresponding author. All authors, including Giuseppe Rengo and Gennaro Pagano, have subsequently agree that they did not qualify for authorship. The remaining authors apologize for any inconvenience caused.
